# Retraction Note: Interactions between Th1 cells and Tregs affect regulation of hepatic fibrosis in biliary atresia through the IFN-γ/STAT1 pathway

**DOI:** 10.1038/s41418-019-0428-0

**Published:** 2019-10-07

**Authors:** Jie Wen, Ying Zhou, Jun Wang, Jie Chen, Wenbo Yan, Jin Wu, Junkai Yan, Kejun Zhou, Yongtao Xiao, Yang Wang, Qiang Xia, Wei Cai

**Affiliations:** 1 Department of Pediatric Surgery, Xin Hua Hospital, School of Medicine, Shanghai Jiao Tong University, Shanghai, China; 2 Shanghai Key Laboratory of Pediatric Gastroenterology and Nutrition, Shanghai, China; 3 Shanghai Institute of Pediatric Research, Shanghai, China; 4 Department of Liver Surgery, Ren Ji Hospital, School of Medicine, Shanghai Jiao Tong University, Shanghai, China

**Retraction Note to: Cell Death and Differentiation**

10.1038/cdd.2017.31

The editors wish to retract this article after publication concerns were raised with respect to the western blot data presented in Fig. 5a, b. The authors have not been able to provide the original, un-cropped, high-resolution images for examination by the editors; the editors therefore no longer have confidence in the integrity of these data. In addition, Fig. 4a, c contain duplicate images of the siSTAT1 samples. Eleven of the twelve authors agree with this retraction. Qiang Xia did not respond to correspondence about this retraction.

